# President’s Address1Presented at the Fourteenth Annual Meeting of the Iowa State Veterinary Medical Association, Des Moines, Ia., February 11-12, 1902.

**Published:** 1902-07

**Authors:** P. O. Koto

**Affiliations:** Forest City, Iowa


### PRESIDENT'S ADDRESS.[1]

By P. O. Koto, M.D.C.,
FOREST CITY, IOWA.

Fellow Members of the Iowa State Veterinary Medical Association:

It is with a feeling of mingled pleasure and regret that I meet with you on this occasion in the capacity of your president. A pleasure, because I have the privilege of associating with you in a great and good work; regret, because of my inability to render you the service you deserve.

I need not dwell upon the significance and importance attached to the annual meeting of this association. I trust that your deliberations and labors will be in the interest of the profession and for its betterment. We come from all parts of this noble and beautiful State, not noted for its snow-clad mountain peaks, nor is its horizon bounded by visions of desert plain or dreary waste; but the sun lifts its first beams over fields of tasselling corn, crimson clover, and velvety blue grass, at its meridian sheds a tropical warmth over a modern Eden, and sinks in splendor over the happy homes and fertile fields of the best agricultural State in the Union. Iowa can boast with pride that she is the garden spot of the universe. Her people are the most intelligent, prosperous, contented and happy. She ranks first among the States in the number of schools, teachers and pupils—in proportion to population. In material resources and all branches of advancement and progress, Iowa is in the fore rank. In stock raising and breeding she can show the best specimens. At the exhibit or on the turf usually from Iowa come the prize winners. This fact greatly emphasizes the importance of the profession of veterinary medicine in Iowa.

That this is an age of progress is self-evident. Especially is this an age of progress in veterinary medicine. Never was the attention of the scientist so turned to investigation of problems directly in the line of advancement of knowledge of veterinary science. The brightest minds of this and foreign countries find congenial occupation in this line of work, although oftentimes of conflicting opinions. Especially is this true of tuber-

[1] Presented at the Fourteenth Annual Meeting of the Iowa State Veterinary Medical Association, Des Moines, Ia., February 11-12, 1902.

culosis, the bone of contention, problems in connection with which, through researches and experiments, will no doubt be satisfactorily solved. Both branches of medical science are to-day fairly launched upon the sea of investigation, striving to unravel the obscure and oftentimes mysterious causation of disease. In the earlier days the members of the veterinary profession were almost entirely those having little or no education. All these things have changed during the last few years. The illiteracy and ignorance once enveloping veterinary science have given way to a most advanced and perfected knowledge, and we are to-day a body of professional scientists ready and capable to assume the responsibilities belonging to this noble calling. Our eligibility to mingle in society on terms of equality with other reputable professions is no longer questioned. The value of the veterinarian not only for the prevention and spread of contagious diseases among domestic animals, but for the valuable assistance rendered toward the preservation of human health, is now undisputed.

We are not unmindful of the fact that much good has been accomplished through the hearty co-operation of the medical profession, and it is a well-recognized fact that the two professions go hand-in-hand, carrying on a combined work for the complete and accurate understanding of those diseases communicable from animal to man. It is said man is the highest of earthly beings; next in physical organization come the domestic animals, such as the horse, the dog, the cow, the hog and the sheep. The men who devote their lives to the care of the health of these animals deserve the same gratitude extended to the sister profession. The successful veterinarian of to-day must be a man of sympathy and kindness, a keen observer of the laws of nature and capable of studying the instincts of the animal. In the practice of veterinary medicine dependence on rules laid down in text-books is not sufficient. I will venture to say that nine out of every ten graduates must earn their living as general veterinary practitioners; they must keep abreast of the theories and discoveries of the passing day. Many graduates of the leading colleges, with no practical training and lack of natural ability, when placed in everyday practice make a complete failure, being utterly incapable of applying their theory to practical purposes.

With the rapid increase in value of horses and other live stock, and the steady increase in value of all farm products, the solid financial condition of the country, and the steady advance in price of real estate, the veterinarian comes in for his share of the prosperity which our country has been blessed with during the last year or more. From what information I can obtain, the services of the veterinarian have been more eagerly sought for by the farmers and stockmen during the last two years than at any other time since the depreciation in live stock which occurred about 1893 and 1894.

The value of a veterinarian as a sanitarian is now better understood. The future of our profession in relation to meat- and milk-inspection is as plain as it is inevitable; our sanitary laws and regulations will be increased and strengthened in harmony with the needs of civilized people.

Regarding the tuberculosis sensation, there are very conflicting opinions among eminent men in favor of and against Professor's Koch's recent theories. The distinguished professor has made the startling announcement that there is nothing in common between human and bovine tuberculosis, while other eminent scientists stoutly deny this. The results of these investigations will be watched with interest by all interested in this great problem.

Allow me to touch briefly on the question of legislation. During our meeting of 1900 it became evident in the minds of the members of our association that an effort should be made to secure legislation. Fortunately, or unfortunately, your humble servant, being a member of the Twenty-eighth General Assembly, was made to feel it his duty, on account of the urgent appeals of the members of the association, to introduce a bill governing veterinary practice in Iowa. With the advice and cooperation of the other members of the committee on legislation a bill was prepared that was satisfactory to that committee and nearly all members of the profession. I will not burden you with the details, but suffice it to say that the bill passed the House without much opposition and passed the Senate after some amendment. Like every great movement, there has to be some individual sacrifice made, but the sacrifices made for the common good of the profession have strengthened the ties of friendship and mutual respect such as nothing else could do.

The many knotty problems predicted have presented themselves, and I want to congratulate the members of the profession for the very able manner and good judgment the Veterinary Board has shown in dealing with the duties pertaining to the new law. Some individuals have been rejected from lack of proper qualifications, and no doubt some who were ineligible have, through misrepresentation, been admitted. I am sorry to be obliged to mention that some members of the profession are inclined to hold the board or the author of the bill responsible for these unavoidable conditions. The profession should rise above criticism when members discharge their duty in aiding the profession. One's duty lies not in tearing down what has already been achieved, but rather in making it stronger. Look at the great industries of the land. What is it that gives them their great power and influence? First and last, it is organization, bound together in mutual interest, without which it would be like a barrel without hoops. In conclusion permit me to say that we should strive to correct what we believe to be wrong. Be honest with yourself and your fellow practitioners, and your profession will go on in the grand triumphal march of prosperity.

Now, gentlemen, this is your meeting. Trusting that you will throw dull care to the dogs and get all the good you can and all the enjoyment possible, I thank you most sincerely for the honor conferred upon me and for the courtesy shown me during my term as president of your association.

